# PNI-Based Nomograms to Predict Tumor Progression and Survival for Patients with Unresectable Hepatocellular Carcinoma Undergoing Transcatheter Arterial Chemoembolization

**DOI:** 10.3390/jcm12020486

**Published:** 2023-01-06

**Authors:** Kai Lei, Zhuo-Fan Deng, Jia-Guo Wang, Ke You, Jie Xu, Zuo-Jin Liu

**Affiliations:** Department of Hepatobiliary Surgery, The Second Affiliated Hospital of Chongqing Medical University, Chongqing 404606, China

**Keywords:** hepatocellular carcinoma (HCC), nomogram, prognostic nutritional index (PNI), survival, transcatheter arterial chemoembolization (TACE), tumor progression

## Abstract

Background: The relationship between the prognostic nutritional index (PNI) and the prognosis of malignancy has been increasingly mentioned in recent research. This study aimed to construct nomograms based on the PNI to predict tumor progression and survival in patients with unresectable hepatocellular carcinoma (HCC) undergoing transcatheter arterial chemoembolization (TACE). Materials and Methods: The development set included 785 patients who underwent their first TACE between 2012 and 2016, and the validation set included 336 patients who underwent their first TACE between 2017 and 2018. The clinical outcomes included the time to progression (TTP) and overall survival (OS). Cox regression was applied to screen for independent risk factors of TTP and OS in the development set, and PNI-based nomograms were constructed for TTP and OS. The predictive performance of nomograms was conducted through the C-index, calibration curves, and decision analysis curves in the development set and validation set. Results: After multivariate analysis, the prognostic predictors of both TTP and OS included portal vessel invasion, extrahepatic metastasis, tumor number, alpha-fetoprotein (AFP) level, longest tumor diameter, and PNI. Furthermore, the Child–Pugh classification and platelets (PLTs) were independent risk factors for OS only. Nomograms for predicting TTP and OS were constructed using TTP and OS prognostic factors. In the development set and the validation set, the C-index of the TTP nomograms was 0.699 (95% confidence interval (CI): 0.680–0.718) and 0.670 (95%CI: 0.638–0.702), and the C-index of the OS nomograms was 0.730 (95%CI: 0.712–0.748) and 0.700 (95%CI: 0.665–0.723), respectively. Conclusion: Nomograms based on the PNI can effectively predict tumor progression and survival in patients with unresectable HCC undergoing TACE.

## 1. Introduction

Although the survival rate of cancer patients has increased, liver cancer is still a major problem. Liver cancer is the sixth most common cancer worldwide and is the second most common cause of cancer death. HCC accounts for a proportion of 75–85% of liver cancer [1,2]. Liver transplantation and radical resection are preferred treatments for early HCC. For HCC patients in relatively advanced stages, interventional embolization therapy, targeted therapy, and immunotherapy are more appropriate treatments [3,4,5]. For unresectable HCC patients with portal vein invasion and extrahepatic metastases, as an effective local treatment modality, TACE can control tumor growth and has a significant efficacy [6]. However, some patients show short-term tumor progression after receiving TACE. Therefore, to receive greater benefits from the TACE treatment modalities, it is important to solve the problem of how to better select appropriate TACE patients.

At present, the BCLC stage, a commonly used stage for HCC patients, can effectively judge the prognosis of patients [7]. However, only a small number of factors are considered for the BCLC stage, and some other factors related to the prognosis of HCC patients, including AFP level, longest tumor diameter, presence of cirrhosis, and platelet count, are ignored [8,9,10].

A growing number of studies have described the immune and nutritional status of cancer patients as an important prognostic factor. As an index associated with the albumin level and the total lymphocyte count, the prognostic nutritional index (PNI) was first mentioned in 1984 [11]. The PNI reflects the immune and nutritional status of patients with malignancy and was proven to be closely associated with the poor prognosis of multiple malignancies, including gastric cancer, intrahepatic cholangiocarcinoma, esophageal squamous cell carcinoma, and nasopharyngeal carcinoma [12,13,14,15]. Meanwhile, the PNI was also shown to be associated with the prognosis of HCC patients [16,17,18]. However, the prognostic role of the PNI in unresectable HCC patients undergoing TACE was not mentioned in these related studies. The purpose of this study was to investigate the role of the PNI in predicting the prognosis of patients with unresectable HCC undergoing TACE, and constructing nomograms by basing the PNI on other independent risk factors to predict tumor progression and long-term survival of unresectable HCC patients.

## 2. Materials and Methods

### 2.1. Patients

This study included 1121 patients who were diagnosed with HCC by histology or imaging and underwent their first TACE between January 2012 and December 2018. The development set included 785 patients who underwent their first TACE between 2012 and 2016, and validation set included 336 patients who underwent their first TACE between 2017 and 2018. All patients met the following inclusion criteria: (1) Barcelona Clinic Liver Cancer (BCLC) stage B or C; (2) preoperative liver function in addition to Child–Pugh class A or B; (3) no other malignancies or other serious underlying diseases; (4) Eastern Cooperative Oncology Group score (ECOG) ≤ 2; (5) regular follow-up; and (6) complete clinical baseline data. Ethics approval was obtained from the ethics committee for this retrospective study. Before receiving any treatment, all patients were informed of the risk, and written informed consent was obtained from patients. The study protocol conforms to the ethical guidelines of the 1975 Declaration of Helsinki (6th revision, 2008) as reflected in the a priori approval from the institution’s human research committee.

### 2.2. Data Collection and Follow-Up

The clinical baseline data of patients included age, sex, stage of BCLC, Child–Pugh classification, portal vessel invasion, extrahepatic metastasis, tumor number, AFP level, longest tumor diameter, alanine aminotransferase (ALT), aspartate transferase (AST), total bilirubin (TBIL), albumin (ALB), hemoglobin (HB), PLT, prothrombin time (PT), and PNI. PNI = serum albumin level (g/L) + 0.005 × total lymphocyte count (per mm^3^). Three to four weeks after the procedure, contrast-enhanced computed tomography (CT) or magnetic resonance imaging (MRI) was used to assess whether patients had residual viable lesions. A re-TACE procedure was carried out if patients still had residual viable lesions. The Response Evaluation Criteria in Solid Tumors (RECIST) was used to classify tumor response. Progressive disease (PD) was defined as tumor progression after the first TACE. Clinical outcomes included TTP and OS. Follow-up by telephone, outpatient visit, and inpatient medical records was provided for all patients after initial treatment. The deadline for follow-up was June 2022. The time between the first TACE and tumor progression, or the time between the first TACE and the follow-up deadline if tumor progression did not occur, was defined as TTP. The time between the first TACE and death, or the time between the first TACE and the follow-up deadline if death did not occur, was defined as OS.

### 2.3. Transcatheter Arterial Chemoembolization Procedure

The Seldinger technique was used to puncture the right femoral artery. After the puncture, a short guide wire was inserted into the 5 Fr micropuncture catheter sheath. Then, under X-ray fluoroscopy, a 5 Fr angiography catheter was inserted into the abdominal trunk and common hepatic artery so that the distribution of tumor vessels could be visualized by angiography. Through the use of a micro-guide wire, a 2.7 Fr angiographic microcatheter was selectively inserted into the tumor nutrient artery and was used to inject a chemotherapeutic agent. The protocol included pirarubicin (20 mg) and iodized oil (5–20 mL). After embolization with iodized oil emulsion, the microsphere diameter was selected based on the size of the tumor and the supply of blood. Five minutes after drug injection, DSA (digital subtraction angiography) was used to confirm that the arterial flow, which provides nutrients to the tumor, was stagnant.

### 2.4. Statistical Analysis

Continuous numerical variables that had a normal distribution were described with the mean value (standard deviation (SD)). Continuous numerical variables that did not have a normal distribution were described with the median (interquartile (IQR)). Counts and percentages were used to summarize categorical variables. Between the development set and validation set, continuous numerical variables were compared by using either the independent samples t-test or the Mann–Whitney U test, and categorical variables were compared with the chi-square test. Univariate and multivariate cox regression analyses were applied to screen for independent risk factors of TTP and OS, and nomogram plots were constructed to predict TTP and OS. The calculation of the C-index was used to assess the predictive power of the models. The difference between the predicted survival rate of the models and the actual survival rate was observed by generating calibration curves of 3-month, 6-month, and 1-year TTP and 1-, 3-, and 5-year OS. The decision curve analysis (DCA) was performed to compare the net benefit and clinical utility of the predictive model and the stage of BCLC. The threshold for the model-predicted probability was used as the x-axis of the analysis curve, and the clinical net benefit was based on the threshold that served as the y-axis of the analysis curve. All statistical analyses were performed by using SPSS 26.0 software (IBM, Chicago, IL, USA) and R software version 4.2.1 (http://www.r-project.org/, accessed on 25 September 2022). *p* ≤ 0.05 was considered statistically significant during the statistical analysis.

## 3. Results

### 3.1. Patient Characteristics

The development set included 785 patients, and the validation set included 336 patients. The baseline data for patients in the development set and validation set are presented in Table 1. There were no significant differences between the development and validation sets in most comparisons of baseline data, including for sex, stage of BCLC, Child–Pugh classification, portal vessel invasion, extrahepatic metastasis, tumor number, AFP level, AST, TBIL, ALB, HB, PLT, and PNI. For age (*p*-value = 0.006), longest tumor diameter (*p*-value = 0.040), ALT (*p*-value = 0.001), and PT (*p*-value = 0.008), there were significant differences between the development set and validation set. The PNI in the development set and the validation set was 44.25 (8.83) and 44.05 (8.29), respectively.

### 3.2. Construction of Predictive Models

**Figure 1:**
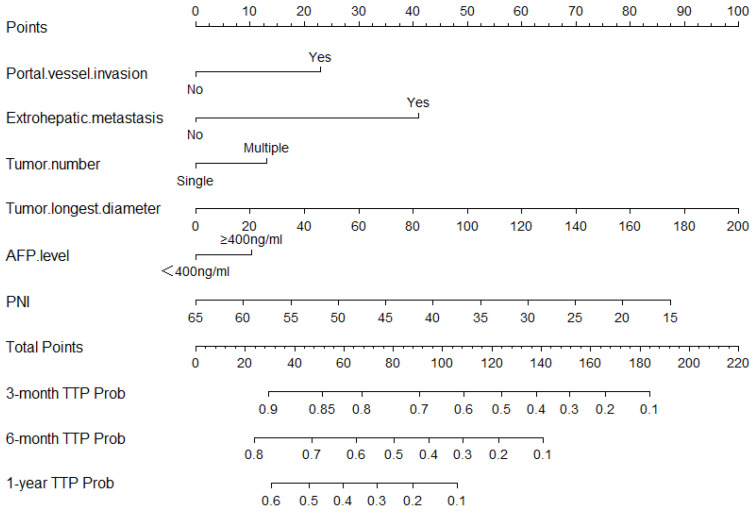
Nomogram for predicting TTP based on PNI. TTP—time to progression; PNI—prognostic nutritional index; AFP—alpha-fetoprotein.

**Figure 2:**
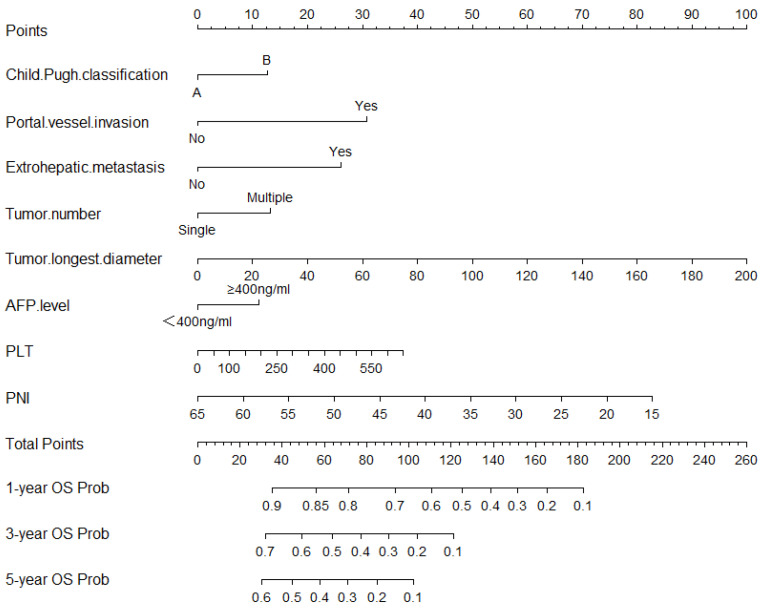
Nomogram for predicting OS based on PNI. OS—overall survival; PNI—prognostic nutritional index; AFP—alpha-fetoprotein.

Independent risk factors for TTP and OS were screened out by using univariate and multivariate Cox regression analyses on the development set (Table 2 and Table 3). ALB and the stage of BCLC were not included in the multivariate analysis due to multicollinearity. After the multivariate Cox regression analysis, six variables were found to be associated with TTP (*p*-value ≤ 0.05), and eight variables were found to be associated with OS (*p*-value ≤ 0.05). The independent prognostic factors associated with TTP were portal vessel invasion (HR = 1.592; 95%CI: 1.304, 1.942; *p* < 0.001), extrahepatic metastasis (HR = 2.268; 95%CI: 1.678, 3.066; *p* < 0.001), tumor number (HR = 1.332; 95%CI: 1.074, 1.653; *p* = 0.009), AFP level (HR = 1.246; 95%CI: 1.067,1.456; *p* = 0.006), longest tumor diameter (HR = 1.010; 95%CI: 1.007, 1.013; *p* < 0.001), and PNI (HR = 0.976; 95%CI: 0.962, 0.990; *p* = 0.001). The independent prognostic factors associated with OS were Child–Pugh classification (HR = 1.268; 95%CI: 1.001,1.604; *p* = 0.049), portal vessel invasion (HR = 1.913; 95%CI: 1.561,2.333; *p* < 0.001), extrahepatic metastasis (HR = 1.720; 95%CI: 1.268, 2.333; *p* < 0.001), tumor number (HR = 1.341; 95%CI: 1.071, 1.679; *p* = 0.01), AFP level (HR = 1.268; 95%CI: 1.074,1.498; *p* = 0.005), longest tumor diameter (HR = 1.011; 95%CI: 1.008, 1.013; *p* < 0.001), PLT (HR = 1.001; 95%CI: 1.000, 1.002; *p* = 0.03), and PNI (HR = 0.969; 95%CI: 0.955,0.984; *p* < 0.001). The 3-month, 6-month, and 1-year nomograms, which were constructed with six independent risk factors associated with TTP to predict TTP, are shown in Figure 1, and the 1-year, 3-year, and 5-year nomograms, which were constructed with independent risk factors associated with OS to predict OS, are shown in Figure 2.

### 3.3. Predictive Ability of Models

The C-index of the TTP nomogram that was constructed for the development set was 0.699 (95%CI: 0.680–0.718), and the C-index of the OS nomogram for the development set was 0.730 (95%CI: 0.712–0.748). In the validation set, the C-index obtained from the constructed TTP nomogram and the OS nomogram was 0.670 (95%CI: 0.638–0.702) and 0.700 (95%CI: 0.665–0.723), respectively. The C-index of the TTP and the OS nomograms proved that the models possessed good accuracy for the prediction of TTP and OS.

**Figure 3:**
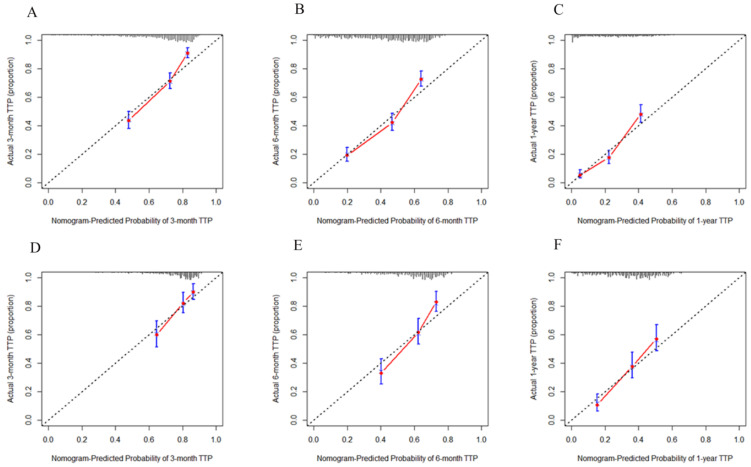
Calibration curves for predicting 3-month, 6-month, and 1-year TTP rates of patients with unresectable HCC after TACE for the development set (**A**–**C**) and the validation set (**D**–**F**). TTP—time to progression; HCC—hepatocellular carcinoma; TACE—transcatheter arterial chemoembolization.

**Figure 4:**
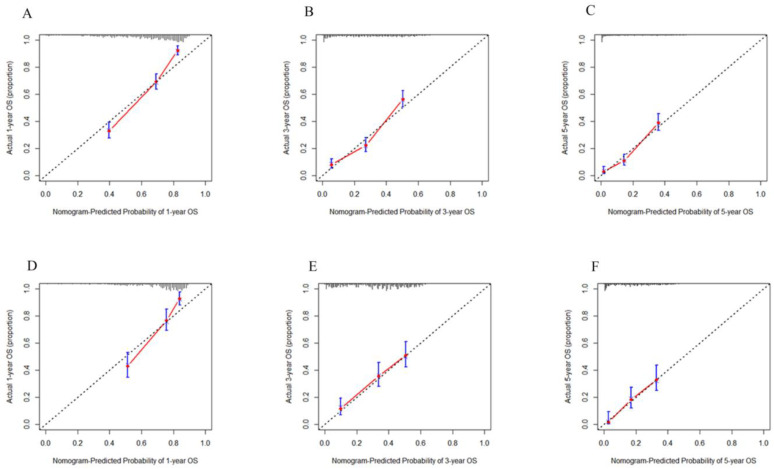
Calibration curves for predicting 1-, 3-, and 5-year OS rates of patients with unresectable HCC after TACE for the development set (**A**–**C**) and the validation set (**D**–**F**). OS—overall survival; HCC—hepatocellular carcinoma; TACE—transcatheter arterial chemoembolization.

The 3-month, 6-month, and 1-year calibration curves of the TTP nomogram for the development set (Figure 3A–C) and validation set (Figure 3D–F) confirmed the best agreement between the prediction of tumor progression and the actual tumor progression. The 1-year, 3-year, and 5-year calibration curves of the OS nomogram for the development set (Figure 4A–C) and validation set (Figure 4D–F) showed that the predicted survival probability was close to the actual survival probability.

**Figure 5:**
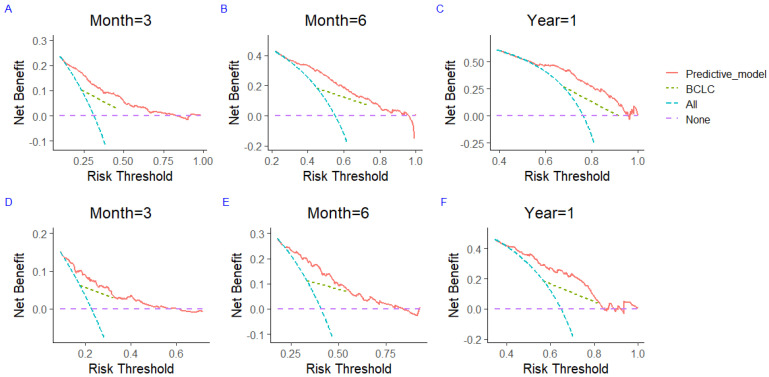
The decision curve evaluates the 3-month, 6-month, and 1-year clinical benefit of the TTP prediction model and compares the clinical benefit of the nomogram prediction model with the clinical benefit of the stage of BCLC for the development set (**A**–**C**) and the validation set (**D**–**F**). TTP—time to progression; BCLC—Barcelona Clinic Liver Cancer.

**Figure 6:**
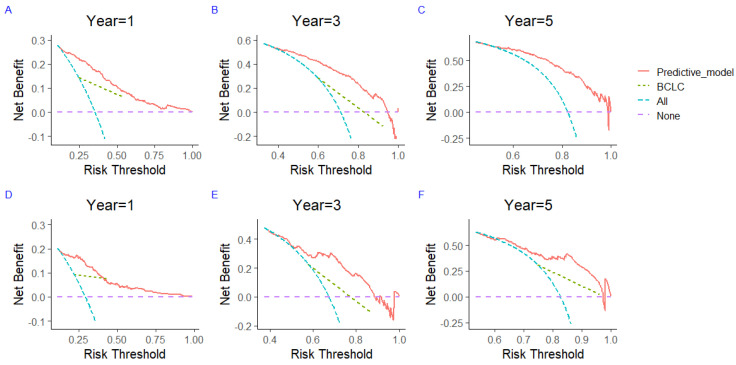
The decision curve evaluates the 1-, 3-, and 5-year clinical benefit of the OS prediction model and compares the clinical benefit of the nomogram prediction model with the clinical benefit of the stage of BCLC for the development set (**A**–**C**) and the validation set (**D**–**F**). OS—overall survival; BCLC—Barcelona Clinic Liver Cancer.

To assess the potential clinical utility of nomograms for predicting TTP and OS, the DCA was performed to compare the net benefit and clinical utility of the predictive model and the stage of BCLC. For the development set (Figure 5A–C) and the validation set (Figure 5D–F), the 3-month, 6-month, and 1-year DCA showed that the TTP model had a greater area than the stage of BCLC. Meanwhile, the 1-year, 3-year, and 5-year DCA of the OS model for the development set (Figure 6A–C) and the validation set (Figure 6D–F) indicated that the TTP model and OS model can obtain a greater clinical net benefit compared to the stage of BCLC.

## 4. Discussion

TACE is a better option for patients with advanced HCC [19]. However, how to select patients suitable for TACE treatment is an important problem for clinicians. This study aimed to explore the significance of the PNI for the prognosis of unresectable HCC patients after receiving TACE treatment and construct PNI-based nomograms to predict tumor progression or survival.

The PNI is based on measurements of serum albumin and the total lymphocyte count [20]. As a component of the PNI, serum albumin is produced by the liver parenchyma. Serum albumin can reflect reserves of the liver function, and it has important physiologic functions such as antioxidant effects, endothelial stabilization, immunomodulation, and other molecule effects [21]. Most HCC patients have a background of hepatitis [22], and the presence of an inflammatory response can reduce serum albumin levels. Meanwhile, the level of serum albumin in HCC is also closely related to the parameters of tumor number, tumor size, and portal vein invasion [23]. Therefore, low serum albumin might indicate a poor prognosis in HCC patients. As another component of the PNI, lymphocytes are also indispensable components of the immune system. Lymphocytes mainly include T-lymphocytes (T-cells) and B-lymphocytes (B-cells). B-cells control tumors by secreting cytokines and T-cells are transformed into cytotoxic T-cells (CTL) to eradicate the tumor cells. Thus, a reduction in lymphocytes implies a decreased ability of the immune system to achieve tumor control and shows the tumor’s invasive ability [24,25]. Some studies proved that a reduced preoperative lymphatic count might predict a poor prognosis in HCC patients [26,27]. Based on serum albumin and total lymphocyte counts, the PNI represents the state of immunity and nutrition. A low PNI means that patients have a poor immune and nutritional status, which may be related to the poor prognosis of tumor patients [12,13,14,15,28]. Meanwhile, the PNI has also been confirmed to be associated with the development and progression of multiple chronic diseases [29,30,31]. Several studies confirmed that the PNI is also closely related to the prognosis of HCC patients [32,33].

This study constructed prognostic models based on the PNI and combined them with clinical data from HCC patients. Independent risk factors for TTP and OS were screened by using a Cox regression analysis. The stage of BCLC and serum albumin levels were excluded from this study during the multivariable Cox regression analysis. As two of the factors considered in the stage of BCLC, extrahepatic metastasis and portal vessel invasion showed multicollinearity with the stage of BCLC in the multivariate regression analysis. Serum albumin also had multicollinearity with the PNI. Independent risk factors for both TTP and OS included portal vessel invasion, extrahepatic metastasis, tumor number, AFP level, longest tumor diameter, and PNI. On the other hand, the independent risk factors for OS included Child–Pugh classification and PLT. Based on previous studies, the cutoff value of the AFP level was set to 400 ng/mL [34]. As a specific marker of HCC, AFP was closely linked with tumor progression and survival of HCC patients [35,36]. Other studies also found that the variables such as tumor number, tumor size, and vascular invasion were independent risk factors for the poor prognosis of HCC patients [37,38]. Nomograms for predicting TTP and OS were constructed by using these independent risk factors to predict tumor progression and survival of unresectable HCC patients after receiving TACE.

This study has the following advantages: firstly, this study was the first time that PNI-based nomograms were established to predict the prognosis of patients with unresectable HCC after receiving TACE. The nomograms in this study were also combined with clinical baseline data, which were easily accessible. The data did not affect the predictive power of the model. Secondly, this study constructed nomograms for TTP and OS to predict tumor progression and survival, respectively. The C-index, calibration curves, and decision analysis curves demonstrated that nomograms of TTP and OS had good predictive power. Unlike other grading systems, the constructed nomograms clearly showed the predicted 3-month, 6-month, and 1-year TTP and the 1-year, 3-year, and 5-year OS of unresectable HCC patients undergoing TACE. Thirdly, by using the decision analysis curves, the constructed models could be visually compared with the stage of BCLC. Through this comparison, it was seen that the constructed nomograms could achieve a greater net clinical benefit.

Some limitations still exist in this study. Firstly, this was a single-center, retrospective study, and the proposed nomogram needs to be externally validated through multicenter studies. Secondly, this study had a small sample size; thus, a large sample size and prospective studies are still needed for validation. Moreover, the tumor microenvironment could affect the function of T-lymphocytes, which might affect the results of this study [39]. Finally, the constructed nomograms in this study were only applied to cases of unresectable HCC patients who underwent TACE, and it is unclear whether they are applicable to predict the prognosis of other HCC patients.

## 5. Conclusions

In this study, nomograms were constructed based on the PNI. These nomograms were visually effective and practical for predicting tumor progression and survival in patients with unresectable HCC undergoing TACE.

## Figures and Tables

**Table 1:** General characteristics of development set and validation set.

Variables		Development Set (*n* = 785)	Validation Set (*n* = 336)	*p*-Value
Age (years) Mean (SD)		53.54 (11.21)	55.52 (10.94)	0.006
Sex	Male	701 (89.3%)	295 (87.8%)	0.464
	Female	84 (10.7%)	41 (12.2%)	
Stage of BCLC	B	483 (61.5%)	214 (63.7%)	0.494
	C	302 (38.5%)	122 (36.3%)	
Background Liver disease	Hepatitis B	612 (78.0%)	272 (81.0%)	0.532
	Hepatitis C	87 (11.1%)	32 (9.5%)	
	Other	86 (11.0%)	32 (9.5%)	
Child–Pugh classification	A	654 (83.3%)	273 (81.3%)	0.403
	B	131 (16.7%)	63 (18.2%)	
Portal vessel invasion	No	531 (67.6%)	224 (66.7%)	0.749
	Yes	254 (32.4%)	112 (33.3%)	
Extrahepatic metastasis	No	733 (93.4%)	322 (95.8%)	0.109
	Yes	52 (6.6%)	14 (4.2%)	
Tumor number	Single	143 (18.2%)	53 (15.8%)	0.324
	Multiple	642 (81.8%)	283 (84.2%)	
AFP level	<400 ng/ml	486 (61.9%)	221 (65.8%)	0.220
	≥400 ng/ml	299 (38.1%)	115 (34.2%)	
Longest tumor diameter (mm) median (IQR)		48 (52)	41 (52)	0.040
ALT (U/L) median (IQR)		41 (39)	37 (29)	0.001
AST (U/L) median (IQR)		48 (42)	46 (39)	0.168
TBIL (umol/L) median (IQR)		15.9 (11.0)	15.8 (11.8)	0.857
ALB (g/L) mean (SD)		38.4 (5.6)	37.7 (5.5)	0.051
HB (g/L) mean (SD)		132 (21)	131 (22)	0.542
PLT (10^9^/L) median (IQR)		111 (95)	105 (99)	0.344
PT (S) median (IQR)		14.3 (1.7)	14.4 (1.8)	0.008
PNI median (IQR)		44.25 (8.83)	44.05 (8.29)	0.720
Progression	No	15 (1.9%)	3 (0.7%)	0.214
	Yes	770 (98.1%)	333 (99.3%)	
TTP (months) median (IQR)		5.0 (9.0)	7.9 (13.2)	<0.001
Status	Survival	114 (14.5%)	47 (14.0%)	0.815
	Death	671 (85.5%)	289 (86.0%)	
OS (months) median (IQR)		18.4 (33.9)	24.1 (32.7)	0.010

BCLC—Barcelona Clinic Liver Cancer; AFP—alpha-fetoprotein; ALT—alanine aminotransferase; AST—aspartate transferase; TBIL—total bilirubin; ALB—albumin; HB—hemoglobin; PLT—platelet; PT—prothrombin time; PNI—prognostic nutritional index; TTP—time to progression; OS—overall survival; SD—standard deviation; IQR—interquartile range.

**Table 2:** Univariate and multivariate analyses of TTP in the development set.

Variables	Univariate Analysis		Multivariate Analysis	
	HR (95%CI)	*p*-Value	HR (95%CI)	*p*-Value
Age (years)	1.000 (0.993, 1.006)	0.922		
Sex (female)	1.120 (0.891, 1.407)	0.332		
Stage of BCLC (C)	2.219 (1.911, 2.576)	<0.001		
Child–Pugh classification (B)	1.428 (1.180, 1.728)	<0.001	1.195 (0.946, 1.509)	0.135
Portal vessel invasion (yes)	2.213 (1.894, 2.586)	<0.001	1.592 (1.304, 1.942)	<0.001
Extrahepatic metastasis (yes)	3.218 (2.415, 4.286)	<0.001	2.268 (1.678, 3.066)	<0.001
Tumor number (multiple)	0.650 (0.540, 0.781)	<0.001	1.332 (1.074, 1.653)	0.009
AFP (≥400 ng/mL)	1.584 (1.368, 1.834)	<0.001	1.246 (1.067, 1.456)	0.006
Longest tumor diameter (mm)	1.013 (1.011, 1.015)	<0.001	1.010 (1.007, 1.013)	<0.001
ALT (U/L)	1.001 (1.000, 1.002)	0.171		
AST (U/L)	1.002 (1.001, 1.002)	<0.001	1.000 (0.999, 1.001)	0.859
TBIL (umol/L)	1.000 (0.997, 1.003)	0.875		
ALB (g/L)	0.964 (0.952, 0.976)	<0.001		
HB (g/L)	0.995 (0.991, 0.998)	0.002	0.996 (0.992, 1.000)	0.079
PLT (10^9^/L)	1.002 (1.001, 1.003)	<0.001	1.000 (0.999, 1.001)	0.584
PT (S)	1.008 (0.970, 1.047)	0.703		
PNI	0.966 (0.956, 0.977)	<0.001	0.976 (0.962, 0.990)	0.001

TTP—time to progression; HR—hazard ratio; CI—confidence interval; BCLC—Barcelona Clinic Liver Cancer; AFP—alpha-fetoprotein; ALT—alanine aminotransferase; AST—aspartate transferase; TBIL—total bilirubin; ALB—albumin; HB—hemoglobin; PLT—platelet; PT—prothrombin time; PNI—prognostic nutritional index.

**Table 3:** Univariate and multivariate analyses of OS in the development set.

Variables	Univariate Analysis		Multivariate Analysis	
	HR (95%CI)	*p*-Value	HR (95%CI)	*p*-Value
Age (years)	0.995 (0.989, 1.002)	0.192		
Sex (female)	1.071 (0.834, 1.375)	0.590		
Stage of BCLC (C)	2.822 (2.410, 3.303)	<0.001		
Child–Pugh classification (B)	1.458 (1.193, 1.782)	<0.001	1.268 (1.001, 1.604)	0.049
Portal vessel invasion (yes)	2.750 (2.337, 3.237)	<0.001	1.913 (1.561, 2.345)	<0.001
Extrahepatic metastasis (yes)	2.965 (2.213, 3.974)	<0.001	1.720 (1.268, 2.333)	<0.001
Tumor number (multiple)	0.562 (0.464, 0.680)	<0.001	1.341 (1.071, 1.679)	0.010
AFP (≥400 ng/mL)	1.681 (1.440, 1.963)	<0.001	1.268 (1.074, 1.498)	0.005
Longest tumor diameter (mm)	1.015 (1.013, 1.017)	<0.001	1.011 (1.008, 1.013)	<0.001
ALT (U/L)	1.001 (1.000, 1.002)	0.154		
AST (U/L)	1.002 (1.001, 1.002)	<0.001	1.000 (0.999, 1.001)	0.418
TBIL (umol/L)	1.001 (0.998, 1.005)	0.396		
ALB (g/L)	0.957 (0.944, 0.970)	<0.001		
HB (g/L)	0.994 (0.990, 0.998)	0.001	0.998 (0.993, 1.002)	0.282
PLT (10^9^/L)	1.003 (1.002, 1.004)	<0.001	1.001 (1.000, 1.002)	0.030
PT (S)	1.012 (0.972, 1.054)	0.548		
PNI	0.962 (0.951, 0.973)	<0.001	0.969 (0.955, 0.984)	<0.001

OS—overall survival; HR—hazard ratio; CI—confidence interval; BCLC—Barcelona Clinic Liver Cancer; AFP—alpha-fetoprotein; ALT—alanine aminotransferase; AST—aspartate transferase; TBIL—total bilirubin; ALB—albumin; HB—hemoglobin; PLT—platelet; PT—prothrombin time; PNI—prognostic nutritional index.

## Data Availability

The datasets used and analyzed during the current study are available from the corresponding author on reasonable request.

## References

[1] Bray F., Ferlay J., Soerjomataram I., Siegel R.L., Torre L.A., Jemal A. (2018). Global cancer statistics 2018: GLOBOCAN estimates of incidence and mortality worldwide for 36 cancers in 185 countries. CA Cancer J. Clin..

[2] Siegel R.L., Miller K.D., Jemal A. (2019). Cancer statistics, 2019. CA Cancer J. Clin..

[3] Llovet J.M., Real M.I., Montaña X., Planas R., Coll S., Aponte J., Ayuso C., Sala M., Muchart J., Solà R. (2002). Arterial embolisation or chemoembolisation versus symptomatic treatment in patients with unresectable hepatocellular carcinoma: A randomised controlled trial. Lancet.

[4] Forner A., Reig M., Bruix J. (2018). Hepatocellular carcinoma. Lancet.

[5] Villanueva A. (2019). Hepatocellular Carcinoma. N. Engl. J. Med..

[6] Lencioni R., de Baere T., Soulen M.C., Rilling W.S., Geschwind J.-F.H. (2016). Lipiodol transarterial chemoembolization for hepatocellular carcinoma: A systematic review of efficacy and safety data. Hepatology.

[7] Forner A., Reig M., de Lope C.R., Bruix J. (2010). Current Strategy for Staging and Treatment: The BCLC Update and Future Prospects. Semin. Liver Dis..

[8] Fujiwara N., Friedman S.L., Goossens N., Hoshida Y. (2018). Risk factors and prevention of hepatocellular carcinoma in the era of precision medicine. J. Hepatol..

[9] Hiraoka A., Michitaka K., Kumada T., Izumi N., Kadoya M., Kokudo N., Kubo S., Matsuyama Y., Nakashima O., Sakamoto M. (2019). Prediction of Prognosis of Intermediate-Stage HCC Patients: Validation of the Tumor Marker Score in a Nationwide Database in Japan. Liver Cancer.

[10] Li S.-P., Cao D., He J.-H., Lou M.-G., Tu X.-X., Li Y. (2022). High platelet count predicts poor prognosis in HCC patients undergoing TACE: A propensity score-matched analysis. Expert Rev. Gastroenterol. Hepatol..

[11] Onodera T., Goseki N., Kosaki G. (1984). Prognostic nutritional index in gastrointestinal surgery of malnourished cancer patients. Nihon Geka Gakkai Zasshi.

[12] Yang Y., Gao P., Song Y., Sun J., Chen X., Zhao J., Ma B., Wang Z. (2016). The prognostic nutritional index is a predictive indicator of prognosis and postoperative complications in gastric cancer: A meta-analysis. Eur. J. Surg. Oncol. EJSO.

[13] Zhang C., Wang H., Ning Z., Xu L., Zhuang L., Wang P., Meng Z. (2016). Prognostic nutritional index serves as a predicative marker of survival and associates with systemic inflammatory response in metastatic intrahepatic cholangiocarcinoma. OncoTargets Ther..

[14] Feng J., Chen Q.-X. (2013). Significance of the prognostic nutritional index in patients with esophageal squamous cell carcinoma. Ther. Clin. Risk Manag..

[15] Yang L., Xia L., Wang Y., Hong S., Chen H., Liang S., Peng P., Chen Y. (2016). Low Prognostic Nutritional Index (PNI) Predicts Unfavorable Distant Metastasis-Free Survival in Nasopharyngeal Carcinoma: A Propensity Score-Matched Analysis. PLoS ONE.

[16] Schütte K., Tippelt B., Schulz C., Röhl F.-W., Feneberg A., Seidensticker R., Arend J., Malfertheiner P. (2014). Malnutrition is a prognostic factor in patients with hepatocellular carcinoma (HCC). Clin. Nutr..

[17] Chan A., Chan S., Wong G.L.-H., Wong V.W.S., Chong C., Lai P.B.S., Chan H.L.Y., To K.-F. (2015). Prognostic Nutritional Index (PNI) Predicts Tumor Recurrence of Very Early/Early Stage Hepatocellular Carcinoma After Surgical Resection. Ann. Surg. Oncol..

[18] Müller L., Hahn F., Mähringer-Kunz A., Stoehr F., Gairing S., Foerster F., Weinmann A., Galle P., Mittler J., dos Santos D.P. (2021). Refining Prognosis in Chemoembolization for Hepatocellular Carcinoma: Immunonutrition and Liver Function. Cancers.

[19] Raoul J.-L., Forner A., Bolondi L., Cheung T.T., Kloeckner R., de Baere T. (2018). Updated use of TACE for hepatocellular carcinoma treatment: How and when to use it based on clinical evidence. Cancer Treat. Rev..

[20] Sun K., Chen S., Xu J., Li G., He Y. (2014). The prognostic significance of the prognostic nutritional index in cancer: A systematic review and meta-analysis. J. Cancer Res. Clin. Oncol..

[21] Spinella R., Sawhney R., Jalan R. (2016). Albumin in chronic liver disease: Structure, functions and therapeutic implications. Hepatol. Int..

[22] Triolo M., Della Corte C., Colombo M. (2014). Impact of HBV therapy on the incidence of hepatocellular carcinoma. Liver Int..

[23] Carr B.I., Guerra V. (2017). Serum Albumin Levels in Relation to Tumor Parameters in Hepatocellular Carcinoma Patients. Int. J. Biol. Markers.

[24] Sanmamed M.F., Chen L. (2019). A Paradigm Shift in Cancer Immunotherapy: From Enhancement to Normalization. Cell.

[25] Man Y.-G., Stojadinovic A., Mason J., Avital I., Bilchik A., Brücher B., Protic M., Nissan A., Izadjoo M., Zhang X. (2013). Tumor-Infiltrating Immune Cells Promoting Tumor Invasion and Metastasis: Existing Theories. J. Cancer.

[26] Yao W., He J.-C., Yang Y., Wang J.-M., Qian Y.-W., Yang T., Ji L. (2017). The Prognostic Value of Tumor-infiltrating Lymphocytes in Hepatocellular Carcinoma: A Systematic Review and Meta-analysis. Sci. Rep..

[27] Chen T.-M., Lin C.-C., Huang P.-T., Wen C.-F. (2012). Neutrophil-to-lymphocyte ratio associated with mortality in early hepatocellular carcinoma patients after radiofrequency ablation. J. Gastroenterol. Hepatol..

[28] Zakosek M., Bulatovic D., Pavlovic V., Filipovic A., Igic A., Galun D., Jovanovic D., Sisevic J., Masulovic D. (2022). Prognostic Nutritional Index (PNI) and Neutrophil to Lymphocyte Ratio (NLR) as Predictors of Short-Term Survival in Patients with Advanced Malignant Biliary Obstruction Treated with Percutaneous Transhepatic Biliary Drainage. J. Clin. Med..

[29] Song W.-J., Li N.-C., Gao J., Xu Z.-P., Liu J.-Y., Long Z., He L.-Y. (2022). The Predictive Significance of Prognostic Nutritional Index and Serum Albumin/Globulin Ratio on the Overall Survival of Penile Cancer Patients Undergoing Penectomy. Curr. Oncol..

[30] Mureșan A.V., Hălmaciu I., Arbănași E.M., Kaller R., Arbănași E.M., Budișcă O.A., Melinte R.M., Vunvulea V., Filep R.C., Mărginean L. (2022). Prognostic Nutritional Index, Controlling Nutritional Status (CONUT) Score, and Inflammatory Biomarkers as Predictors of Deep Vein Thrombosis, Acute Pulmonary Embolism, and Mortality in COVID-19 Patients. Diagnostics.

[31] Kaller R., Arbănași E.M., Mureșan A.V., Voidăzan S., Arbănași E.M., Horváth E., Suciu B.A., Hosu I., Halmaciu I., Brinzaniuc K. (2022). The Predictive Value of Systemic Inflammatory Markers, the Prognostic Nutritional Index, and Measured Vessels’ Diameters in Arteriovenous Fistula Maturation Failure. Life.

[32] Man Z., Pang Q., Zhou L., Wang Y., Hu X., Yang S., Jin H., Liu H. (2018). Prognostic significance of preoperative prognostic nutritional index in hepatocellular carcinoma: A meta-analysis. HPB.

[33] Pinato D.J., North B.V., Sharma R. (2012). A novel, externally validated inflammation-based prognostic algorithm in hepatocellular carcinoma: The prognostic nutritional index (PNI). Br. J. Cancer.

[34] Yang S.-L., Liu L.-P., Ren J.-W., Fang X., Chen G.G., Lai P.B.S. (2016). Preoperative serum α-fetoprotein and prognosis after hepatectomy for hepatocellular carcinoma. Br. J. Surg..

[35] Watanabe T., Tokumoto Y., Joko K., Michitaka K., Horiike N., Tanaka Y., Tada F., Kisaka Y., Nakanishi S., Yamauchi K. (2021). AFP and eGFR are related to early and late recurrence of HCC following antiviral therapy. BMC Cancer.

[36] Mehta N., Heimbach J., Harnois D.M., Sapisochin G., Dodge J.L., Lee D., Burns J.M., Sanchez W., Greig P.D., Grant D.R. (2017). Validation of a Risk Estimation of Tumor Recurrence After Transplant (RETREAT) Score for Hepatocellular Carcinoma Recurrence After Liver Transplant. JAMA Oncol..

[37] Wang L., Jin Y.-X., Ji Y.-Z., Mu Y., Zhang S.-C., Pan S.-Y. (2020). Development and validation of a prediction model for microvascular invasion in hepatocellular carcinoma. World J. Gastroenterol..

[38] Xu X.-F., Xing H., Han J., Li Z.-L., Lau W.-Y., Zhou Y.-H., Gu W.-M., Wang H., Chen T.-H., Zeng Y.-Y. (2019). Risk Factors, Patterns, and Outcomes of Late Recurrence After Liver Resection for Hepatocellular Carcinoma: A Multicenter Study from China. JAMA Surg..

[39] Verma N.K., Wong B.H.S., Poh Z.S., Udayakumar A., Verma R., Goh R.K.J., Duggan S.P., Shelat V.G., Chandy K.G., Grigoropoulos N.F. (2022). Obstacles for T-lymphocytes in the tumour microenvironment: Therapeutic challenges, advances and opportunities beyond immune checkpoint. eBioMedicine.

